# Condom use prevalence during the COVID-19 pandemic among female sex workers in Dakar, Senegal: a retrospective, cross-sectional analysis

**DOI:** 10.1093/heapol/czaf023

**Published:** 2025-04-07

**Authors:** Wen Qiang Toh, Carole Treibich, Sandie Szawlowski, Henry Cust, Elhadj A Mbaye, Khady Gueye, Cheikh T Ndour, Aurélia Lépine

**Affiliations:** Erasmus School of Economics, Erasmus University Rotterdam, Burgemeester Oudlaan 50, Rotterdam, PA 3062, The Netherlands; University Grenoble Alpes, CNRS, INRAE, Grenoble INP, GAEL, Grenoble 38000, France; Institute for Global Health, University College London, 30 Guilford Street, London WC1N 1EH, United Kingdom; Global Health and Development Department, London School of Hygiene & Tropical Medicine, Keppel Street, London WC1E 7HT, United Kingdom; HIV/STI Division, Ministry of Health and Social Action of Senegal, Rue Aimee Cesaire, Dakar 4024, Senegal; HIV/STI Division, Ministry of Health and Social Action of Senegal, Rue Aimee Cesaire, Dakar 4024, Senegal; HIV/STI Division, Ministry of Health and Social Action of Senegal, Rue Aimee Cesaire, Dakar 4024, Senegal; Institute for Global Health, University College London, 30 Guilford Street, London WC1N 1EH, United Kingdom

**Keywords:** COVID-19, HIV, sexually transmitted infections, sex workers, LMIC, risky sexual behaviours

## Abstract

Literature suggests that individuals may trade off health for income in face of an economic shock. Being in a close contact profession, the livelihoods of sex workers were severely affected by the COVID-19 pandemic. Few studies exist on whether prevalence of better-renumerated condomless sex increased among this population in low and middle-income countries and discuss its implications on HIV/STI transmission, specifically during pandemic situations. We reported cross-sectional condom use prevalence estimates of 600 female sex workers in Dakar, Senegal from data collected before (2015, 2017) and during the pandemic (June–July 2020). Condom use prevalence was elicited via list experiments for more truthful estimates. Double list experiment estimates of mean condom use prevalence declined from 78.2% (95% CI: 70.9–85.5%) in 2017 to 65.1% (95% CI: 57.6–72.7%) in 2020. This statistically significant decrease of 13.1 percentage points (*P* = .014) represents a 16.8% fall in condom use and a 60.2% increase in condomless sex prevalence. The fall in condom use prevalence was largely concentrated amongst the asset-poor, providing some suggestive evidence that economic reasons drove the fall in condom use, reinforcing findings in existing literature regarding the positive relationship between economic shocks and risky sexual behaviours. At the point of the survey, the observed decline in client numbers exceeded the reduction in condom use prevalence, suggesting potential mitigation of HIV/STI transmission risks during the COVID-19 pandemic; nevertheless, the lack of direct comparability between these two metrics warrants cautious interpretation. However, more accurate epidemiological modelling considering the non-sex worker population and longer-term studies on whether condom use prevalence returned to pre-COVID levels after client numbers recovered are required for a comprehensive assessment of the pandemic’s short-term and longer-term impact on HIV/STI transmission.

Key messages

- Condom use prevalence among sex workers in Dakar, Senegal dropped significantly during the COVID-19 pandemic.
- There is suggestive evidence that this was driven by economic shocks, as the drop was significant among those with fewer assets.
- The drop in client numbers was steeper than the drop in condom use prevalence, mitigating short-term HIV/STI risk.
- This study suggests further research into whether economic measures may potentially be useful levers in HIV/STI reduction.

## Introduction

The low rate of recorded COVID-19 infections in Africa (WHO 2020) has been partly attributed to the quick response of African governments in implementing stringent countermeasures to curtail the spread of COVID-19 (Binagwaho 2020). Despite the benefits of a low infection rate, there have been concerns of extensive unintended consequences from COVID-19 mitigating measures (Turcotte-Tremblay et al. 2021). With widespread poverty and non-existent financial safety nets, the burden of such restrictions is likely to disproportionately fall on the most vulnerable groups. There is evidence in the literature that economic shocks increased risky sexual behaviours, potentially constituting one of the key drivers of HIV/AIDS (Burke et al. 2015, Cust et al. 2020).

Sex workers are particularly vulnerable in this context. The high frequency and close contact nature of sex work and social distancing measures implemented—such as night curfews and closure of entertainment venues where they solicit business—suggest a significant loss of work and income. Under these circumstances, there may be strong economic incentives for sex workers to engage in condomless sex as a coping mechanism against this economic shock since condomless sex tends to be much better remunerated (Rao et al. 2003, Gertler et al. 2005, de la Torre et al. 2010, Muravyev and Talavera 2018, Quaife et al. 2019). Studies have found that condomless sex prevalence among sex workers in sub-Saharan Africa increased during civil conflicts and when faced with illness in the family (Robinson and Yeh 2011, Dupas and Robinson 2012). Despite being marginalized and often overlooked in government provisions, sex workers play a significant role in HIV/STI transmission. Economic factors continue to heavily influence sexual practices among female sex workers (FSWs), as recent studies illustrate. For instance, an unpublished study by Cust et al. (2024) in Cameroon found that FSWs receive 30% higher compensation for condomless sex acts, underscoring the economic pressures driving risky behaviours. Similarly, research by Lépine and co-authors Lépine et al. (2024) in Cameroon highlights how economic incentives can undermine safer sex practices. These findings demonstrate that economic constraints and incentives for risky behaviours remain persistent barriers to HIV prevention, underscoring the need for interventions that address the economic realities faced by FSWs.

In Senegal, where we carried out our study, HIV prevalence in the general population is low, but is concentrated amongst FSWs (Lépine and Treibich 2020). In 2015, HIV/AIDS prevalence among FSWs was 6.6%, nine times that of the HIV prevalence among the general population (APAPS & IRESSEF 2016). This elevated prevalence underscores the critical role that commercial sex plays as a major contributor to HIV transmission in Western Africa (Alary and Lowndes 2004, Côté et al. 2004, Lowndes et al. 2002. The COVID-19 pandemic added new challenges to this already vulnerable population. Senegal, one of the first African countries to detect a COVID-19 case in March 2020 (WHO Regional Office of Africa 2020), responded rapidly with measures including a nationwide night curfew. While the curfew was progressively shortened, bars and nightclubs—prime venues for soliciting clients, especially for legally registered FSWs—remained closed for an extended period (Sall 2020) (refer to Appendix A for more information). The resulting economic hardship forced many FSWs to adapt, often by engaging in riskier sexual practices, potentially exacerbating HIV/STI transmission and creating a significant public health issue as a collateral effect of the pandemic.

Our objectives in this paper were two-fold. First, using a measure of condom use less prone to strategic misreporting, we show whether condom use prevalence has declined during the COVID-19 pandemic among FSWs in Dakar, Senegal and show suggestive evidence of factors correlated with this decline. Second, we discuss the short and longer-term potentials of the COVID-19 pandemic to trigger a HIV/STI public health crisis.

## Materials and methods

### Survey design

From 18 May to 6 July 2015, we conducted a survey of 654 FSWs in Dakar, Senegal, representing 15% of the estimated total FSW population in the city APAPS (2014). This study was carried out with strong involvement from local stakeholders and community members to ensure effective engagement and representativeness. Collaboration with the DLSI under the Ministry of Health facilitated access to four of the five STI health centres in Dakar where registered FSWs receive services. These health centres provided invaluable support in identifying and recruiting registered FSWs through midwives in charge of the registration services. For unregistered FSWs, who are often harder to reach, we partnered with peer leaders—sex workers with deep knowledge of their communities—and non-governmental organization staff working with unregistered FSWs. Peer leaders played a critical role in identifying and recruiting participants, ensuring that the sampling strategy was community-based and grounded in trust, which is essential for working with vulnerable populations. In Senegal, sex work is legal for those who are formally registered, which informed the dual approach of accessing participants through formal health services and grassroots networks of peer leaders. This combined strategy captured diverse experiences and perspectives within the FSW population and reflects our commitment to community engagement and leveraging existing structures to address the challenges of reaching and representing key populations in research.

Convenience sampling was employed due to the challenges of accessing this population, with recruitment criteria requiring participants to be at least 18 years old and actively engaged in sex work. Given the often-blurred line between transactional sex and commercial sex, we employed a rigorous identification process to distinguish between the two. For registered FSWs, we utilized official registration records from health centres accessed through our collaboration with the DLSI. For unregistered FSWs, recruitment was facilitated through peer leader networks. To confirm participants’ active engagement in commercial sex, all individuals were asked at the start of each interview whether they were currently earning income from sex work. This approach ensured a clear differentiation between sex work and other forms of transactional sex, enhancing the precision and validity of our sample.

We used the same methodology to recruit participants from 7 to 26 August 2017 and from 29 June to 28 July 2020. For both survey waves, we sought participation of former survey respondents. They were contacted using the telephone number they declared in the previous wave they participated, or if uncontactable, we relied on peer FSWs and midwives to reach them. The survey attrition rate was around 30% for each of these waves. The minimum detectable effect (MDE) for a binary outcome with a baseline proportion of 50%, a sample size of 600, 80% power, and a 5% significance level is ∼8.1 percentage points. An MDE of this size is both a critical indicator for policymakers to act upon and an intriguing finding for public health researchers seeking to understand and address the unintended consequences of global health pandemics. To meet the recruitment target of 600 in both years, each cohort was replenished with new survey participants.

In 2015 and 2017, surveys were conducted in private rooms in four out of the five STI health centres in Dakar. In 2020, the survey was held at external venues near the health centres to minimise the risks of COVID-19 infection to staff and participants. COVID-19 preventive measures taken can be found in Appendix B.

Each interviewer-led structured survey lasted 1.5 h, where a broad range of research questions were investigated. Ethical clearance for each of the survey waves was obtained from the national ethics committee in Senegal and from the PI’s university in 2015, 2017, and 2020, respectively. An information sheet about the survey objectives and how their data would be protected was given to participants and written consent was sought before the commencement of the survey. Survey participants were reimbursed for their transport costs and the time spent at the health facility.

### Final analysis sample

**Figure 1.**
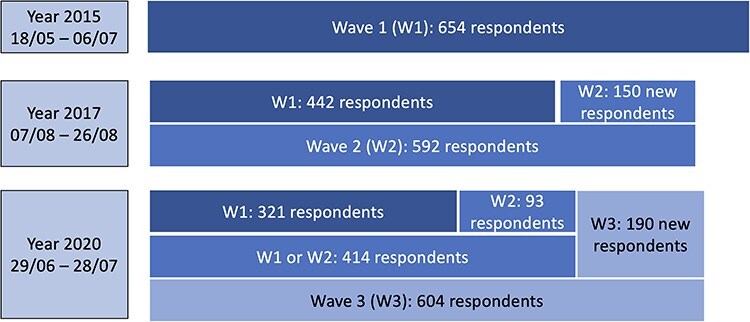
Survey attrition and sample replenishment.

**Figure 2.**
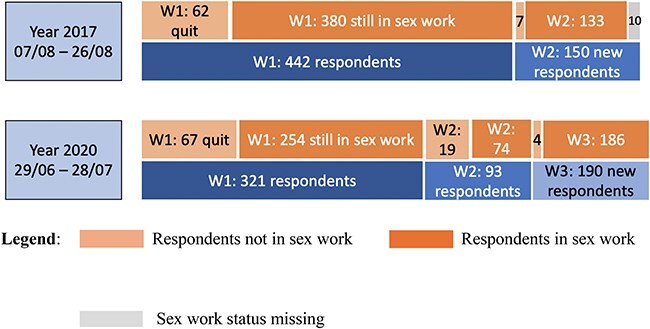
Sex work status of respondents.

The analyses in this paper were constrained to respondents who were still in sex work in each survey year. This comprised 654, 513, and 514 respondents in 2015, 2017, and 2020, respectively (Figure 1). The lower numbers in the latter 2 years reflected a lower survey recruitment target as well as a sex work quit rate of 14.0–20.8% (69 out of 442 and 86 out of 414 repeat survey respondents had quit sex work as of the 2017 and the 2020 survey, respectively.) between survey waves characteristic of this population (Figure 2).

### Elicitation of condom use via list experiments

The use of direct self-reports in condom use in a face-to-face interview has been found to severely over-estimate condom use prevalence among FSWs in Senegal by 19 percentage points due to social desirability bias (Treibich and Lépine 2019).

A list experiment is a well-established method in the literature for truthful elicitation (Imai 2011, Blair and Imai 2012, Glynn 2013, Blair et al. 2018, Lépine et al. 2020). It randomizes respondents into two groups. Each respondent reports the total number of correct statements in a list containing some non-sensitive statements. In the treatment group, the list contains one additional sensitive statement. The mean difference of the total count of statements between the two groups reflects the proportion of respondents who agreed with the sensitive statement. In our context, the sensitive statement was ‘I used a condom during my last intercourse with a client’ (Table 1). The recall period for the list experiment was defined as ‘last sex’ rather than a longer recall period or a frequency-based measure. This decision was made for several reasons. First, the list experiment method is designed to accommodate binary outcomes, making it unsuitable for categorical variables such as the frequency of condomless sex. Second, focusing on ‘last sex’ reduces the likelihood of misreporting by limiting the recall period to a recent event, which participants are more likely to remember accurately. Third, this approach allows for the control of specific characteristics of the sexual act in question, thereby enhancing the precision and reliability of the data collected.

**Table 1.** Lists in list experiment

List #1		List #2	
Control	Treatment	Control	Treatment
It is safer to bring a client home than going to a hotel.	It is safer to bring a client home than going to a hotel.	The majority of my clients are Senegalese.	The majority of my clients are Senegalese.
I prefer that the client pays me before intercourse.	(I used a condom during my last intercourse with a client.)	I usually spend the whole night with my client	(I used a condom during my last intercourse with a client)
Monday is the day I have the greatest number of clients.	I prefer that the client pays me before intercourse.	I usually solicit clients by phone	I usually spend the whole night with my client
	Monday is the day I have the greatest number of clients.		I usually solicit clients by phone

In 2015, only List #1 was implemented (single list experiment). In 2017 and 2020, List #2 was added. Participants were then randomly assigned to either the treatment group of List #1 or the control group of List #1 (and conversely for List #2) based on their interview order as determined by the enumerator. Specifically, individuals with an even interview number were assigned to List #1, while those with an odd interview number were assigned to List #2. This alternation was implemented automatically within the data entry system, ensuring that neither the enumerator nor the participant could influence the number of items presented. Double list experiment estimates were derived from the joint analysis of both lists, helping to reduce the large standard errors of the condom use prevalence estimates (Lépine et al. 2020). An ordinary least squares regression was used to estimate condom use prevalence between the two groups. Standard errors were clustered at the respondent level.


$${n_{i,l}} = {a_l} + \beta {G_{i,l}} + {e_{i,l}}$$

where *n_i, l_* is the number of statements in List *#l* respondent *i* agrees with. *a_l_* takes the value 1 for List #2, and 0 for List #1. *G_i, l_* takes the value 1 if respondent *i* is shown List *#l* with the sensitive statement, 0 otherwise. *e_i, l_* is the error term. *β* represents condom use prevalence of the last sex act.

The survey instructions are found in Appendix C. Assumptions for the validity of the list experiment method are discussed in Appendix D.

### Analysis of sex work outcomes during the COVID-19 pandemic

We compared how condom use prevalence estimates, monthly sex work earnings and weekly client numbers differed cross-sectionally across the 2015, 2017, and mid-2020 surveys. For earnings and client numbers, the mean, median, interquartile range, 10th and 90th percentile were shown as the outcomes were skewed. We used the two-sided Wilcoxon rank-sum test to test for differences in outcomes across survey waves.

A weakness of comparing outcomes across survey waves is that it risks capturing time trends unrelated to COVID-19. Therefore, we supplemented the analysis by also reporting respondents’ self-reports of how COVID-19 has affected their sex work activities on a Likert-type scale. These self-reports allow us to elicit COVID-19-specific effects.

### Analysis of condom use and economic shocks

Condomless sex is a way for sex workers to boost their income amidst an income shock with limited clients. We expect condom use declines to be steeper amongst the asset poor than the asset rich, as those with few assets are less likely to have the resources to buffer themselves against income shocks. Asset levels were derived by summarizing ownership of assets—such as television, radio, computer, and more—into a wealth index via multiple correspondence analysis (Appendix E:I). Asset poor is defined as having an asset level that is less or equal to the median.

Borrowing could be a way to cope with economic shocks in the shorter term, reducing the need to rely on condomless sex. However, in the longer term, especially in a prolonged crisis, debt accumulation may force debtors to use condomless sex to raise funds for debt repayment. We look at condom use prevalence changes among sex workers whose households are indebted versus debt-free, and the interaction of asset status and debt status.

## Results

### Active FSW sample

Table 2 displays the demographics of active FSW in each wave. Demographics and registration status was relatively stable across waves, except for the 2.5 years increase in average age (0.27 standard deviation) between 2015 and 2017.

**Table 2.** Demographics of active female sex workers by survey wave

	Overall demographics								
	2015			2017			2020		
	*N*	Mean	SD	*N*	Mean	SD	N	Mean	SD
Age	654	35.83	9.25	513	38.34	9.40	514	38.99	9.73
Registered FSW (Prop.)	653	0.50	0.50	512	0.50	0.50	514	0.47	0.50
Never married (Prop.)	654	0.24	0.43	513	0.20	0.40	514	0.21	0.41
Married (Prop.)	654	0.01	0.10	513	0.02	0.14	514	0.01	0.09
Divorced (Prop.)	654	0.69	0.46	513	0.69	0.46	514	0.70	0.46
Widowed (Prop.)	654	0.05	0.22	513	0.09	0.29	514	0.08	0.27

Notes: ‘*N*’ is the number of observations. ‘SD’ means standard deviation. ‘Prop.’ means Proportion.

### Sex work earnings and client numbers

**Figure 3.**
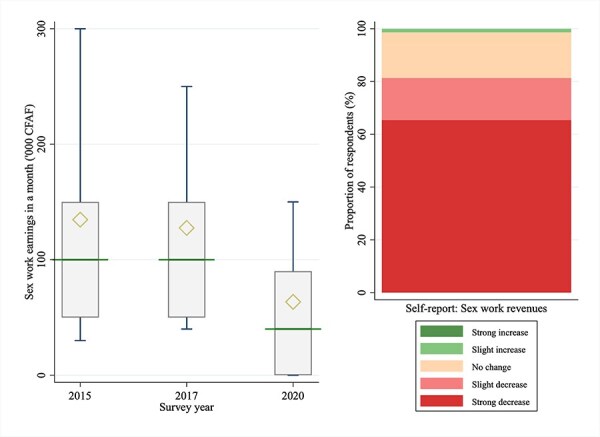
Sex work earnings of FSWs in Dakar, Senegal.

Cross-survey comparisons suggested a substantial decline in monthly sex work earnings in 2020 (Table 3, Fig. 3). Median monthly sex work earnings in local currency (CFA franc) was 100 000 in both 2015 and 2017, but fell by 60.0% to 40 000 in 2020. The two-sided Wilcoxon rank-sum test failed to reject the null hypothesis that the distributions of 2015 and 2017 were similar (*P* = .62), while it rejected the null hypothesis that the distributions of 2015 vis-à-vis 2020 (*P* < .0001) and 2017 vis-à-vis 2020 (*P* < .0001) were similar. Approximately 65% of the respondents reported that their income from sex work has strongly decreased because of COVID-19.

**Table 3.** Comparison of sex work earnings and client numbers across survey waves

										Wilcoxon rank-sum test					
	2015			2017			2020			2015 vs. 2017		2015 vs. 2020		2017 vs. 2020	
	*N*	Median (IQR)	Mean (SD)	*N*	Median (IQR)	Mean (SD)	*N*	Median (IQR)	Mean (SD)	*P*-value^a^	*P* (2015 > >15 )^b^	*P*-value	*P* (2015 > >15 )	*P*-value	*P* (2017 > >17 )
**Sex work earnings in a month**	652	100	134.5	653	100	127.6	623	40	63.4	.62	.508	<.0001	.746	<.0001	.743
**(‘000 CFAF)**		(50–150)	(125.9)		(50–150)	(111.3)		(0–90)	(86.4)						
**Clients in a week**	507	5	6.5	513	6	8.4	512	1	2.5	.0008	.443	<.0001	.807	<.0001	.827
**(No.)**		(3–8)	(6.4)		(3–10)	(8.8)		(0–4)	(4.1)						

Notes: ‘*N*’ stands for the number of observations. ‘IQR’ means the interquartile range and ‘SD’ refers to the standard deviation.

a The *P*-value of the two-sided Wilcoxon rank-sum test is with regards to the null hypothesis that the distribution of the two populations are the same.

b *P* (year 1 > year 2) represents the probability that a random draw from year1 would be larger than a random draw of year 2.

**Figure 4.**
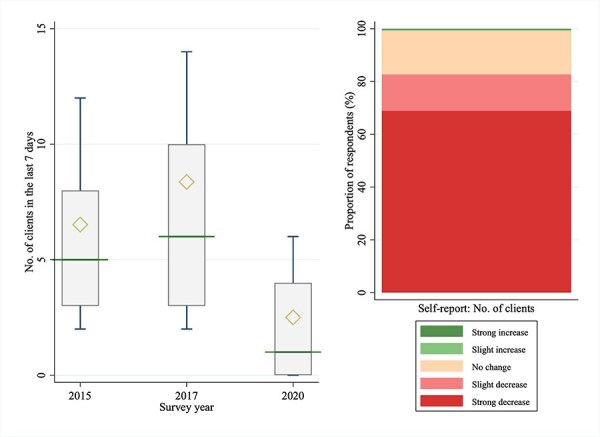
Number of clients of FSWs in Dakar, Senegal.

Client numbers have also fallen drastically (Table 3, Fig. 4). The median number of clients in a week was 5, 6, and 1 in 2015, 2017, and 2020, respectively. The median number of clients in 2020 fell by 83.3% with respect to 2017. The two-sided Wilcoxon rank-sum test rejected the null hypothesis of equal distributions for all pairwise comparisons of survey years. Nearly 69% of respondents reported that COVID-19 has strongly reduced the number of clients.

### Overall condom use prevalence

**Figure 5.**
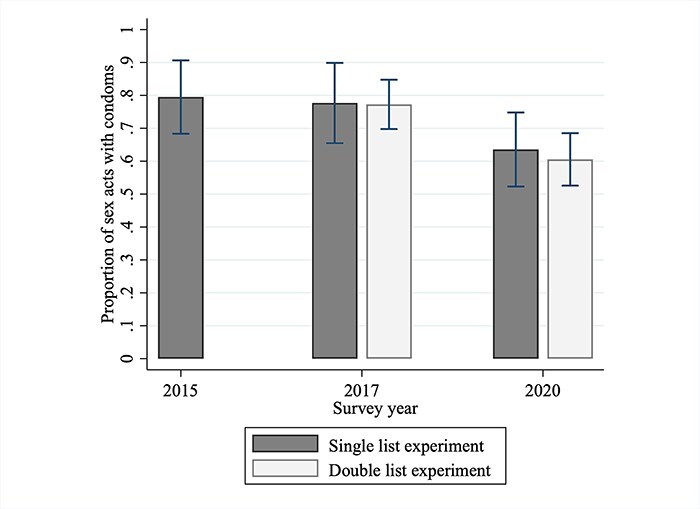
Condom use prevalence estimates of FSWs in Dakar, Senegal.

Condom use prevalence estimates from the single list experiment were very similar in 2015 and 2017. They were 79.6% (95% CI: 68.5–90.8) and 78.0% (95% CI: 66.0–90.0) respectively (Fig. 5). However, in 2020, this fell to 67.9% (95% CI: 57.2–78.6). Due to the large standard errors from analysing a single list, none of the pairwise changes were statistically significant (2015 vs. 2017, *P* = .84; 2015 vs. 2020, *P* = .21; 2017 vs. 2020, *P* = 0.13).

The point estimates from the single and double list experiments corresponded closely with each other in 2017 and 2020, but the latter had smaller standard errors (Fig. 5). Condom use prevalence decreased from 78.2% (95% CI: 70.9–85.5%) in 2017 to 65.1% (95% CI: 57.6–72.7%) in 2020 and this decrease was statistically significant (*P* = .014). This represented a drop of 16.8% in condom use prevalence in 2020, and in terms of condomless sex, a 60.2% increase from 2017.

We did a robustness check on whether this result could be caused by the fact that the sample of respondents differed across the survey waves. We employed entropy balancing, reweighing the 2017 and 2020 samples to match the sample moments of 2015’s age, marital status, sex work registration status, and household size—characteristics that we collected and were unlikely to be affected by COVID-19. This reweighing reduced the differences between the samples across years. Our key result of similar condom prevalence between 2015 and 2017, and a large drop in condom use prevalence in 2020 remained robust in this reweighed sample (Appendix F).

### Condom use prevalence by asset and debt status

#### Asset

Mean condom use prevalence declined by 22.9 percentage points amongst asset-poor FSWs from 2017 and 2020 and this decline was statistically significant (*P* = .004) (Table 4). In percentage terms, this was a 27.2% decline. In contrast, the decline in mean condom use prevalence amongst the asset-rich was non-significant at 4.1 percentage points (*P* = .60), corresponding to a 5.5% decline- around five times smaller than that of asset-poor FSWs. The difference between the two subgroups was not statistically significant at the 5% significance level (*P* = .087); however, the magnitude of the differences is large. These results were robust to reweighing the samples using entropy balance to reduce cross-survey differences (Appendix F, Table F.3).

**Table 4.** Condom use prevalence by asset and debt status

	Double list experiment condom use prevalence estimate (%)								
	2017			2020					
	*N*	mean	SE	*N*	mean	SE	Fall in condom use (pp)		Fall in condom use w.r.t. 2017 (%)
**Asset status**									
Asset poor	213	84.2	(5.7)	280	61.3	(5.2)	22.9	(*P* = .0035)	27.2
Asset rich	236	73.8	(5.6)	234	69.7	(5.7)	4.1	(*P* = .60)	5.5
Difference							18.8	(*P* = .087)	
**Debt status**									
Indebted	263	80.9	(5.3)	280	73.1	(5.2)	7.8	(*P* = .29)	9.7
Not indebted	250	75.4	(5.2)	234	55.6	(5.6)	19.9	(*P* = .0085)	26.3
Difference							−12.0	(*P* = .25)	
**Asset status × Debt status**									
**Asset poor**									
Indebted	110	90.4	8.2	181	70.0	6.5	20.5	(*P* = .047)	22.6
Not indebted	103	77.7	8.2	99	45.7	8.4	32.0	(*P* = .0053)	41.2
Difference							−11.5	(*P* = .44)	
**Asset rich**									
Indebted	115	74.8	8.0	99	79.1	8.4	−4.3	(*P* = .71)	−5.7
Not indebted	121	72.8	7.8	135	62.8	7.6	9.9	(*P* = .35)	13.6
Difference							−14.2	(*P* = .37)	

Notes: Standard errors (SE) were clustered by respondent. The double list experiment was implemented only in 2017 and 2020. Debt status was elicited only in 2017 and 2020. Asset status was elicited only in 2015 and 2020. Asset status in 2017 was first filled with asset status in 2020, and then by asset status in 2015. The reverse fill order was also done as a robustness check, and results were similar.

#### Debt

Slightly >51% of the respondents’ households were indebted in 2017. This increased slightly to 54.5% in 2020, equivalent to a 6.2% increase. Respondents whose households were debt-free in 2020 had a mean condom use prevalence estimate that was 19.9 percentage points lower than those whose households debt-free in 2017, and this decline was statistically significant (*P* = .009) (Table 4). In contrast, the mean condom use prevalence decline was smaller and non-significant amongst FSWs whose households were indebted (7.8 percentage points, *P* = .29). The difference between the two subgroups was large but not statistically significant (*P* = .25). These results were robust to reweighing the samples using entropy balance to reduce cross-survey differences (Appendix F, Table F.3).

#### Asset X Debt

The asset poor whose households are debt-free saw the steepest decline in mean condom use prevalence (32.0 percentage points), and this decline was statistically significant (*P* = .0053) (Table 4). In percentage terms, this was a 41.2% decline from 2017 levels. The asset poor whose households were indebted saw the second steepest decline (20.5 percentage points), and this decline was statistically significant (*P* = .047). In percentage terms, this was a 22.6% decline from 2017 levels, half of that for those with debt-free households.

Amongst the asset rich, there was a non-significant change in mean condom use prevalence regardless of household debt status (*P* = .35, *P* = .71). Among the asset rich, those whose households were debt-free also saw a higher mean condom use prevalence decline compared to those whose households were indebted (14.2 percentage points), although this difference was not statistically significant.

These results were robust to reweighing the samples using entropy balance to reduce cross-survey differences (Appendix F, Table F.3).

## Discussion

### Condom use prevalence and economic shocks

The data suggested that sex workers were severely affected by the COVID-19 pandemic. Sex work income and client numbers decreased substantially in 2020. This observation was corroborated by the respondents’ self-reports. We observed that condom use prevalence has also fallen substantially in 2020 despite being stable between 2015 and 2017. Investigation of the mechanisms behind the fall in condom use prevalence suggests that economic reasons are likely to be a driver. We observed much steeper condom use prevalence declines among the asset-poor FSWs. Even though the difference between subgroups is not statistically significant at the 5% level (*P* = .086), the magnitude of this difference was very large (18.8 percentage points). In addition, the difference in condom use prevalence between 2017 and 2020 were statistically significant only among the asset-poor. This provides suggestive evidence for the economic shock hypothesis.

While differences between the subgroups were not statistically significant, we consistently observed steeper condom use prevalence declines among debt-free households than indebted ones, suggesting that borrowing could have been an alternative way for individuals to tide through the loss in sex work earnings early in the pandemic. One implication of this finding is that gaining access to borrowing or financial assistance could potentially incentivise FSWs— whether during the pandemic or during normal times- to use protection with clients.

### COVID-19 pandemic and short-term versus longer-term HIV/STI transmission risk

The overall amount of condomless sex has likely decreased as the decline in client numbers likely outweighed the increased prevalence of condomless sex. Therefore, HIV/STI transmission risk—at least among sex workers—would likely have fallen. While these figures seem optimistic, we note that the survey in 2020 was carried out just 3–4 months into the COVID-19 pandemic. In the longer term, what is of concern is whether depressed condom use prevalence would persist after client numbers increase again. One possible mechanism could be through excessive debt accumulation during the pandemic. In a prolonged crisis such as COVID-19, borrowing limits and debt repayment may become issues, potentially resulting in future declines in condom use prevalence, were not observed in the time frame of this current study. In addition, excessive debt accumulation during the pandemic may defer the impact of a current economic shock to the future if a substantial amount of debt needs to be repaid quickly after client numbers recover. This effect has been observed in the literature. In a study on the 2-month Kenyan civil conflict, the total number of condomless sex acts initially declined as the decrease in demand for sex dominated the increase in the prevalence of condomless sex (Dupas and Robinson 2012). However, the total number of condomless sex acts eventually increased when demand returned (Dupas and Robinson 2012). Backed by reports from informal qualitative interviews, the authors posited that respondents were motivated to recoup their income losses, and therefore, continued to engage in condomless sex for some time, cautioning that a crisis that lasts longer could create ‘behavioural responses that could have important effects on the spread of HIV and other STIs’ (Dupas and Robinson 2012). Similarly, in our context, a key concern would be whether condom use prevalence would increase back to pre-COVID levels when client numbers improve or whether the economic shock would be propagated in the future. In addition, there is a risk that behavioural norms of condom use may also start changing given that COVID-19 was a prolonged crisis.

There could be other potential mechanisms that may be important in explaining the decline in condom use prevalence. However, most mechanisms imply that condom use prevalence should revert once client numbers improve and hence, may be of limited interest. In Appendix G, we look at some of these, namely risk compensation, risk substitution, and condom supply. We could not find any suggestive evidence that these factors contributed to the condom use decline.

### Policy implications

The findings of our study, which show that negative economic shocks like those induced by COVID-19 have influenced the risky sexual practices of sex workers in Senegal, carry significant implications for policy and programming aimed at preventing HIV. These results underscore the vulnerability of female sex workers to economic instability, highlighting the need for targeted coping strategies that can help mitigate the impact of income shocks on their health behaviour- a result also found by Gong et al. (2019). Policies that provide economic support, such as unconditional cash transfers or income protection schemes, could play a crucial role in reducing risky sexual behaviours by alleviating the financial pressures that drive such practices. Our findings resonate with the recent trial by Lépine et al. (2024) in Cameroon, which demonstrated the effectiveness of such interventions in reducing HIV risks among vulnerable women. By integrating economic safety nets into HIV prevention programming, policymakers can address both the immediate and long-term risks faced by women at risk of HIV, contributing to more sustainable and equitable public health outcomes. Interventions such as unconditional cash transfers or low-burden loaning mechanisms could play a critical role in reducing economic stress and, by extension, lowering the incidence of HIV/STIs among key populations. By providing financial relief, these interventions would allow individuals to make safer health decisions without the added pressure of economic survival. Additionally, these structural interventions could address the broader social determinants of health, fostering resilience within vulnerable communities and promoting long-term health equity.

### Limitations of study

Given the difficulties in conducting studies on FSWs in sub-Saharan Africa, this study has many limitations. First, convenience sampling had to be employed. Peer-to-peer recruitment drove majority of the survey participation as FSWs are a hard-to-reach population. Therefore, cross-survey differences in sample could inhibit cross-survey comparability. The comparison of participants who remained in the study and those who attrited based on observable characteristics did not reveal systematic differences (see Appendix H). While we cannot fully exclude the possibility of unobservable factors influencing attrition, the evidence suggests that the impact of attrition on the results is likely minimal. Furthermore, our robustness checks reweighing the 2017 and 2020 survey sample to match the distribution of characteristics in 2015 survey show that adjusting for cross-survey differences did not affect the key results shown. Second, we only have 2 years of pre-COVID data collection, and the previous wave was held in 2017, which was 3 years before the 2020 survey. With such a short time-series, we cannot eliminate the possibility that time-variant changes or other confounding factors in 2020 could have also contributed to changes in measured condom use prevalence. In Appendix I, we study one of these potential time-varying confounders: Tabaski—a festival which happened just after our survey in 2020 which will also create economic shocks for our respondents. Our key results remained similar, and the magnitude of the decline of condom use was about four-fifths of the unadjusted effect. Fourth, the use of the list experiment was essential for eliciting more truthful condom use prevalence estimates in Senegal. However, the trade-off is the high standard errors from the list experiment estimation. This presents difficulties in comparing between subgroups. In particular, it is important to highlight that the reliability of the list experiment method heavily depends on the careful design of both sensitive and non-sensitive items to minimize measurement error. Additionally, while the Central Limit Theorem supports the robustness of the sample mean comparison, deviations from its assumptions in smaller samples could still lead to understated uncertainties. Future research could explore the extent to which alternative non-parametric methods may complement or validate findings derived from the list experiment approach.

This study does not aim to provide a comprehensive epidemiological assessment, and several critical aspects remain unaddressed. For instance, one important missing piece is the response of vulnerable individuals who are not sex workers but may increase their participation in transactional sex during time of crisis (Robinson and Yeh 2011). Additionally, even among sex workers, HIV/STI transmission risk is affected by a multitude of factors, including shift towards a more regular clientele, network effects, the heterogeneities in behavioural changes, variations in the number of sex acts per client, and increase in riskier practices such as anal sex. A more comprehensive epidemiological modelling will be required to accurately assess the overall impact of the COVID-19 pandemic on HIV/STI transmission risk.

Moreover, Senegal’s legal regulation of sex work—a relatively uncommon approach in the region—introduces unique contextual dynamics that may influence the outcomes observed in this study. Government’s responses to the COVID-19 pandemic also varied greatly across countries, further emphasizing the need for research in diverse settings.

## Conclusions

We observed a decline in condom use prevalence amongst female sex workers during the COVID-19 pandemic, with suggestive evidence that economic reasons could be one of the key drivers. This would imply that policies alleviating economic hardship—such as food vouchers, cash assistance, and microfinance—could be potentially effective levers for reducing risky sexual behaviours. Nonetheless, there is currently still a dearth of research on the effectiveness and value for money such economic interventions as a HIV/STI prevention tool.

The steep fall in client numbers during the COVID-19 pandemic mitigates HIV/STI transmission risk. However, this must be confirmed in more comprehensive epidemiological modelling including women involved in transactional sex. We proposed that whether condom use prevalence would return to pre-COVID levels after client numbers improve would be one key consideration that should be monitored in future pandemics.

## Supplementary Material

czaf023_Supp

## Data Availability

Anonymized data will be published on the university’s public repository after publication.

## Reflexivity statement

This work is a joint collaboration of contributors from Senegalese institutions (authors EM, KG, CN) and European institutions (authors WQT, CT, SS, HC, AL) that span multiple levels of seniority both within Senegalese institutions and European institutions. There is good gender balance—five authors are female and three are male. WQT, CT, SS, HC and CN author specialize in global health and development economics, with CT and AL having substantial past experience on conducting field studies. The fifth has extensive experience in conducting fieldwork among Senegalese sex workers. KG and CN have keen expertise in health and health system especially in the local context in Senegal.
