# Supplementary material for: Condom use prevalence during the COVID-19 pandemic among female sex workers in Dakar, Senegal: a retrospective, cross-sectional analysis
Source: Health Policy Plan. 2025 Apr 7;40(7):685–95. doi: 10.1093/heapol/czaf023 (PMC12360168; doi:10.1093/heapol/czaf023)
Supplement: czaf023_Supp [file czaf023_supp.zip › Appendix.docx]

**Appendix**

# Appendix A: Background context in Senegal Sex work in Senegal

Sex work in Senegal is legal and regulated. Female sex workers (FSWs) must register with the authorities and attend monthly clinic visits to practise legally (Mackintosh, 2020). Being able to practise legally allows FSWs to solicit outdoors- such as in bars and nightclubs- without the fear of fines and arrests. This often entails a higher income than can be sought through more indoor solicitation methods, such as phones and internet. However, many FSWs choose to remain unregistered to avoid exposure of being tied to this highly-stigmatised occupation. 46.2 percent of the sample involved in a national survey on FSWs in Senegal were registered

(APAPS & IRESSEF, 2016), while around half of our survey sample was registered.

# COVID-19 situation in Senegal

Senegal was one of the first African countries to detect a COVID-19 case on 2 March 2020 (WHO Regional Office of Africa, 2020). The Senegalese government responded rapidly by announcing a national state of health emergency. Measures that were implemented include enforcing self-isolation measures, banning public gatherings, closing borders and prohibiting inter-regional travel. On 23 March 2020- within three weeks of the first known case- a night curfew from 8pm to 6am was implemented.

The night curfew implemented on 23 March 2020 was progressively shortened. We carried out a round of data collection from 29 June to 28 July 2020 in the midst of the pandemic. On 30 June 2020 - the second day of our most recent survey wave - the curfew was completely lifted and the state of emergency lifted. Nonetheless, bars and nightclubs- which were prime venues for soliciting clients especially for registered FSWs- remained closed throughout the entire duration of the survey (Sall, 2020). These measures meant that the mode of sex work has also changed, with more indoor solicitation methods (e.g. phone, internet) being used than before.

The COVID-19 pandemic has proven to be a long-drawn crisis. In January 2021, to stall the second wave of COVID-19 infections, Senegal re-implemented the state of emergency and a night curfew. The strict measures adopted in Senegal have thus far successfully limited the number of COVID-19 cases and deaths to 41,416 and 1,139 respectively as at 31 May 2021 (Ritchie et al., 2021) in a population of 16.7 million (UN, 2021). However, the economic and social fallout from the COVID-19 pandemic has been substantial and is touted to be one of the reasons fuelling protests in March 2021.

# References

1. Mackintosh E. (2020, August 31) *Sex work is legal here. But only if you register with the police.* Available from: https://edition.cnn.com/2019/09/20/health/senegal-sex-workasequals-intl/index.html
2. APAPS & IRESSEF (2016). *Enquête nationale de surveillance combinée desinfections sexuallement transmissibles et du VIH/SIDA, Groupe cible: Travailleuses du sexe. Dakar, Senegal.*
3. WHO Regional Office of Africa (2020, March 2). *Le Sénégal déclare le premier cas de COVID-19*. [https://www.afro.who.int/fr/news/le-senegal-declare-le-premier-cas-de-covid-](https://www.afro.who.int/fr/news/le-senegal-declare-le-premier-cas-de-covid-19)

[19](https://www.afro.who.int/fr/news/le-senegal-declare-le-premier-cas-de-covid-19)

1. Sall M. (2020, June 29) *Message à la Nation de SEM le Président Macky SALL: Levée de l’état d’urgence instauré dans le cadre de la lutte contre la maladie à coronavirus COVID-*

*19*. https://sante.sec.gouv.sn/sites/default/files/DISCOURS%20DU%20PR.pdf

1. Ritchie et al. (2021, May 31). *Coronavirus (COVID-19) Cases*. <https://ourworldindata.org/covid-cases>
2. UN (2021, March 31). *Senegal*. <http://data.un.org/en/iso/sn.html>

# Appendix B: COVID-19 protocol in 2020 survey

We were committed to guarantee safety and health of participants and of our team during data collection. When we conducted the survey, there were no published COVID-19 protocols available from the relevant ethics committees. As a result, we develop a strict COVID-19 protocol and added a “staying healthy” module, outlining the COVID-19 regulations, in the trainings of data collectors and supervisors to minimise risks of infection. In addition, interviews were not held in locations where COVID-19 patients were being treated. Two out of four hospitals were treating COVID-19 patients. These survey locations were moved to nearby secure offices.

The protocol enforced is as follows:

- Assessment of any COVID-19 symptoms over the phone before inviting the participant on site
- Provision and mandatory use of facial masks to all participants and members of our team on the survey sites and use of facial masks for members of staff in public places (public transport, market and religious sites) during the whole period of the survey
- Provision and use of hand sanitiser at the beginning and end of the survey by participants and enumerators
- Tablets, used for collecting data, will be cleaned and disinfected after every interview
- Everyone onsite must maintain a six-foot distance
- Daily cleaning and disinfecting of interview rooms
- Establishment of a 14-day quarantine after a known exposure, and isolation and testing before returning to work. Note this was not applied, since no COVID-19 case was reported by participants or the survey team.

# Appendix C: Instructions of list experiment

I [The interviewer] am going to read you three (four) sentences. Please count how many of those sentences you agree with. You do not have to tell me which sentences you agree with, just how many sentences you agree with.

To help you count the number of sentences you agree with, I am going to give you three (four) marbles. Please place these marbles in your right hand and keep your hands behind your back. If you agree with the sentence I am reading, please transfer one marble from your right hand to your left hand. If you do not agree with this sentence, please do nothing. Once all the sentences have been read, you will tell me how many sentences you agree with. This number should correspond to the number of marbles you have in your left hand. I will now read those sentences.

## (A) Lists

### List #1

1. It is safer to bring a client home than going to a hotel.
2. *(I used a condom during my last intercourse with a client.)*
3. I prefer that the client pays me before intercourse.
4. Monday is the day I have the greatest number of clients.

### List #2

1. The majority of my clients are Senegalese.
2. *(I used a condom during my last intercourse with a client)*
3. I usually spend the whole night with my client
4. I usually solicit clients by phone

# Appendix D: Checking assumptions of list experiment

The validity of the list experiment is dependent on certain assumptions, such as no ceiling, floor and design effects. We tested the validity of the list experiment for each survey wave.

## (A) Checking for ceiling and floor effects

The proportion of individuals in the control group answering “0” and “3” should be relatively low. If they are not, the randomised nature of the experiment suggests that this proportion would be similar in the treatment group, which imply that privacy of the respondents’ answers to the sensitive question cannot be ensured.

While for the 2015 and 2017, the assumptions of no ceiling and floor effects seem to be met, in 2020, there is a significant violation in the “no ceiling effect” assumption. In particular, for the second list, a much higher proportion of respondents in the control group answered all three non-sensitive statements affirmatively (23.4%), double of that in 2017.

Looking at the statements in the second list, this change is consistent with how sex work has changed during the COVID-19 pandemic. In particular, we would expect more respondents to agree with the statements that majority of their clients are Senegalese, and that they usually solicit clients by phone than in previous years due to international travel restrictions and the closure of entertainment venues, therefore increasing the mass at the ceiling at “3”.

The privacy of the FSW answers regarding condom use could be violated in the presence of ceiling effects. However, since *unprotected sex* is the undesirable behaviour they are unwilling to report, and the list experiment is designed such that to report *unprotected sex* the FSW agrees with one fewer statements, having a high average number of non-sensitive statements to agree with will not affect the propensity for FSWs to be dishonest in their answers and should not affect our estimates of condom use.

To illustrate, there are two scenarios if a treated respondent were to agree with the three non-sensitive statements:

**Scenario 1: Respondent used a condom with her last client**

There will be no disincentive for such a respondent to report her answer as “4”, as using condoms is a socially-approved behaviour.

**Scenario 2: Respondent did not use a condom with her last client**

Such a respondent may choose to answer “3” truthfully, or misreport her answer as “4”. However, there is a low incentive to misreport, as if she were to answer “3”, it will still be impossible to recover her individual condom use.

In contrast, the absence of floor effects, i.e. a low proportion of respondents should disagree with all non-sensitive statements, is the more crucial assumption. A response of “0” by treated respondents would divulge to others that they did not use a condom- the sensitive behaviour. Therefore, there is a high incentive to misreport at this margin. This absence of floor effects assumption is likely to be met in all years.

### Table D.1: Ceiling and floor effects of list experiment

|  | **Proportion of respondents in control group answering**  **"0" (%) List 1** | **List 2** | **List 1** | **Proportion of respondents in control group answering**  **"3" (%)**  **List 2** |
| --- | --- | --- | --- | --- |
| 2015 | 2.8 | n.a. | 9 | n.a. |
| 2017 | 2.3 | 2.4 | 8.9 | 10.2 |
| 2020 | 0.8 | 0 | 8.3 | 23.4 |

## (B) Checking for design effects

The addition of the sensitive statement should not change a respondent’s answer to non-sensitive statement. The cumulative proportion of treated who agrees with at least *i* statements should be at least that of the controls if so.

### Table D.2: Design effects of list experiment

|  |  |  | **List 1** | |  | **Cumulative proportion of respondents agreeing to at least** |  |  | ***i*** | **statements (%) List 2** |  |  |
| --- | --- | --- | --- | --- | --- | --- | --- | --- | --- | --- | --- | --- |
|  | **i = 0** | **i = 1** | **i = 2** | **i = 3** | **i=4** | **sum(diff)*** | **i = 0** | **i = 1** | **i = 2** | **i = 3** | **i=4** | **sum(diff)** |
| **2015** |  |  |  |  |  |  |  |  |  |  |  |  |
| control | 100 | 97.2 | 63.9 | 8.9 | 0.0 |  |  |  | n.a. |  |  |  |
| treated | 100 | 99.4 | 91.5 | 50.6 | 8.2 |  |  |  | n.a. |  |  |  |
| *difference* | 0 | 2.2 | 27.6 | 41.7 | 8.2 | 79.7 |  |  | n.a. |  |  |  |
| **2017** |  |  |  |  |  |  |  |  |  |  |  |  |
| control | 100 | 97.7 | 57.4 | 8.9 | 0.0 |  | 100 | 97.7 | 81.2 | 10.2 | 0.0 |  |
| treated | 100 | 100 | 92.2 | 44.7 | 5.1 |  | 100 | 99.6 | 96.5 | 61.6 | 9.7 |  |
| difference | 0 | 2.3 | 34.8 | 35.8 | 5.1 | 78.0 | 0 | 1.9 | 15.3 | 51.4 | 9.7 | 78.3 |
| **2020** |  |  |  |  |  |  |  |  |  |  |  |  |
| control | 100 | 99.2 | 64.8 | 8.3 | 0.0 |  | 100 | 100 | 85.1 | 23.4 | 0.0 |  |
| treated | 100 | 99.6 | 96.2 | 41.8 | 2.7 |  | 100 | 99.2 | 95.7 | 66.8 | 9.1 |  |
| *difference* | 0 | 0.4 | 31.4 | 33.5 | 2.7 | 68.0 | 0 | -0.8** | 10.6 | 43.4 | 9.1 | 62.3 |

Notes:

* sum(diff) is the condom use prevalence estimate derived from the list

** While this number should not be negative, 0.8% consists of two observations in the sample. Therefore it is possible that this result could be attributable to random chance.

## (C) Checking randomisation of groups

We tabulated selected variables of each group in each year. Few variables had strong statistical significance, suggesting that overall, the randomisation went reasonably well. Note that the control group in List #1 is the treatment group in List #2, vice versa.

### Table D.3: Differences between control and treatment groups in list experiment

**Variables**

**Control in List #1**

**Treatment in List #1**

**Mean Diff**

|  | **N** | | **mean** | **N** | | **mean** | |  |
| --- | --- | --- | --- | --- | --- | --- | --- | --- |
| **2015** |  | |  |  | |  | |  |
| Age | 324 | | 35.54 | 330 | | 36.12 | | -0.578 |
| Registered FSW (Prop.) | 324 | | 0.475 | 329 | | 0.523 | | -0.0470 |
| Sex work earnings in a month ('000 CFAF) | 322 | | 136.6 | 330 | | 132.6 | | 4.008 |
| Clients in a week (No.) | 323 | | 6.492 | 330 | | 6.530 | | -0.0380 |
| Average price of last two clients ('000 CFAF) | 309 | | 17.86 | 314 | | 15.61 | | 2.253* |
| Total household expenses in last 30 days ('000 CFAF) | 324 | | 338.4 | 330 | | 321.0 | | 17.37 |
| Savings in last 30 days ('000 CFAF) | 319 | | 12.41 | 329 | | 19.54 | | -7.125 |
| Share of occasional clients (Prop.) | 316 | | 0.378 | 322 | | 0.411 | | -0.0320 |
| At least one outdoor solicitation method (Prop.) | 323 | | 0.678 | 328 | | 0.698 | | -0.0200 |
| At least one outdoor place of sex (Prop.) | 323 | | 0.378 | 328 | | 0.372 | | 0.00600 |
| **2017** |  |  | |  |  | |  | |
| Age | 258 | 38.02 | | 255 | 38.67 | | -0.643 | |
| Registered FSW (Prop.) | 258 | 0.488 | | 254 | 0.508 | | -0.0200 | |
| Sex work earnings in a month ('000 CFAF) | 255 | 122.6 | | 252 | 132.6 | | -9.991 | |
| Clients in a week (No.) | 258 | 8.380 | | 255 | 8.325 | | 0.0540 | |
| Average price of last two clients ('000 CFAF) | 258 | 15.12 | | 254 | 18.02 | | -2.898 | |
| Total household expenses in last 30 days ('000 CFAF) | 258 | 335.1 | | 255 | 328.6 | | 6.471 | |
| Savings in last 30 days ('000 CFAF) | 258 | 24.51 | | 255 | 18.80 | | 5.718 | |
| Indebted (Prop.) | 258 | 0.512 | | 255 | 0.514 | | -0.00200 | |
| Share of occasional clients (Prop.) | 254 | 0.338 | | 251 | 0.300 | | 0.0370 | |
| Had STI symptoms with any of the last two clients (Prop.) | 246 | 0.0810 | | 249 | 0.0320 | | 0.049** | |
| At least one outdoor solicitation method (Prop.) | 258 | 0.535 | | 254 | 0.535 | | -0.00100 | |
| At least one outdoor place of sex (Prop.) | 258 | 0.585 | | 254 | 0.610 | | -0.0250 | |
| **2020** |  |  | |  |  | |  | |
| Age | 253 | 38.37 | | 261 | 39.60 | | -1.230 | |
| Registered FSW (Prop.) | 253 | 0.447 | | 261 | 0.490 | | -0.0440 | |
| Sex work earnings in a month ('000 CFAF) | 251 | 61.90 | | 260 | 64.95 | | -3.050 | |
| Clients in a week (No.) | 253 | 2.557 | | 261 | 2.456 | | 0.101 | |
| Average price of last two clients ('000 CFAF) | 253 | 13.53 | | 260 | 14 | | -0.473 | |
| Total household expenses in last 30 days ('000 CFAF) | 253 | 242.2 | | 261 | 226.7 | | 15.44 | |
| Savings in last 30 days ('000 CFAF) | 253 | 14.20 | | 261 | 14.62 | | -0.428 | |
| Indebted (Prop.) | 253 | 0.545 | | 261 | 0.544 | | 0.00100 | |
| Share of occasional clients (Prop.) | 250 | 0.264 | | 260 | 0.309 | | -0.045* | |
| Had STI symptoms with any of the last two clients (Prop.) | 253 | 0.0400 | | 261 | 0.0310 | | 0.00900 | |
| At least one outdoor solicitation method (Prop.) | 253 | 0.419 | | 261 | 0.464 | | -0.0450 | |
| At least one outdoor place of sex (Prop.) | 253 | 0.486 | | 260 | 0.581 | | -0.095** | |

* p<0.1, ** p<0.05, *** p<0.01

# Appendix E: Dimensionality reduction

## I. Multiple correspondence analysis for asset ownership

1 television, 2 radio, 3 video/CD/DVD player, 4 gas/electric stove, 5 improved fireplace, 6 fridge, 7 air-conditioning, 8 computer, 9 landline, 10 mobile phone, 11 washing machine, 12 internet at home, 13 car, 14 moped/motorbike

**Multiple/Joint correspondence analysis N = 1,168**

**Total inertia = 0.0187513**

**Method: Burt/adjusted inertias No. of axes = 2**

|  | **principal inertia** | **percent** | **cumulative percent** |
| --- | --- | --- | --- |
| **dim 1** | 0.0139054 | 74.16 | 74.16 |
| **dim 2** | 0.0013206 | 7.04 | 81.2 |
| **dim 3** | 0.0002232 | 1.19 | 82.39 |
| **dim 4** | 0.0000942 | 0.5 | 82.89 |
| **dim 5** | 7.68E-06 | 0.04 | 82.93 |

**Total** 0.0187513 100

**Statistics for column categories in standard normalization**

| **Categories** | | | **mass** | **overall Quality** | | **%inert** | | **coord** | **dimension_1 sqcorr** | | **contrib** | | **coord** | **dimension_2 sqcorr** | | **contrib** | |
| --- | --- | --- | --- | --- | --- | --- | --- | --- | --- | --- | --- | --- | --- | --- | --- | --- | --- |
| **z1**  0 | | | 0.014 | 0.87 | | 0.045 | | 1.703 | 0.681 | | 0.041 | | 2.914 | 0.189 | | 0.121 | |
| 1 | | | 0.057 | 0.87 | | 0.011 | | -0.424 | 0.681 | | 0.01 | | -0.726 | 0.189 | | 0.03 | |
| **z2**  0 | | | 0.042 | 0.823 | | 0.025 | | 0.752 | 0.718 | | 0.024 | | 0.934 | 0.105 | | 0.037 | |
| 1 | | | 0.029 | 0.823 | | 0.035 | | -1.077 | 0.718 | | 0.034 | | -1.339 | 0.105 | | 0.053 | |
| **z3**  0 | | | 0.063 | 0.881 | | 0.011 | | 0.442 | 0.854 | | 0.012 | | 0.253 | 0.027 | | 0.004 | |
| 1 | | | 0.008 | 0.881 | | 0.084 | | -3.467 | 0.854 | | 0.097 | | -1.987 | 0.027 | | 0.032 | |
| **z4**  0 | | | 0.037 | 0.76 | | 0.027 | | 0.785 | 0.639 | | 0.023 | | 1.11 | 0.121 | | 0.046 | |
| 1 | | | 0.034 | 0.76 | | 0.029 | | -0.861 | 0.639 | | 0.025 | | -1.217 | 0.121 | | 0.05 | |
| **z5**  0 | | | 0.05 | 0.701 | | 0.006 | | 0.301 | 0.565 | | 0.005 | | 0.478 | 0.135 | | 0.011 | |
| 1 | | | 0.022 | 0.701 | | 0.013 | | -0.687 | 0.565 | | 0.01 | | -1.091 | 0.135 | | 0.026 | |
| **z6**  0 | | | 0.053 | 0.853 | | 0.025 | | 0.695 | 0.771 | | 0.026 | | 0.736 | 0.082 | | 0.029 | |
| 1 | | | 0.018 | 0.853 | | 0.073 | | -2.046 | 0.771 | | 0.076 | | -2.167 | 0.082 | | 0.085 | |
| **z7**  0 | | | 0.068 | 0.857 | | 0.003 | | 0.222 | 0.853 | | 0.003 | | -0.053 | 0.005 | | 0 | |
| 1 | | | 0.004 | 0.857 | | 0.053 | | -4.035 | 0.853 | | 0.061 | | 0.965 | 0.005 | | 0.003 | |
| **z8**  0 | | | 0.068 | 0.852 | | 0.007 | | 0.332 | 0.842 | | 0.008 | | -0.121 | 0.011 | | 0.001 | |
| 1 | | | 0.003 | 0.852 | | 0.134 | | -6.725 | 0.842 | | 0.152 | | 2.45 | 0.011 | | 0.02 | |
| **z9**  0 | | | 0.07 | 0.716 | | 0.002 | | 0.151 | 0.616 | | 0.002 | | -0.197 | 0.1 | | 0.003 | |
| 1 | | | 0.002 | 0.716 | | 0.07 | | -5.543 | 0.616 | | 0.058 | | 7.242 | 0.1 | | 0.099 | |
| **z10**  0 | | | 0.005 | 0.336 | | 0.018 | | 0.532 | 0.064 | | 0.002 | | 3.566 | 0.272 | | 0.069 | |
| 1 | | | 0.066 | 0.336 | | 0.001 | | -0.044 | 0.064 | | 0 | | -0.294 | 0.272 | | 0.006 | |
| **z11**  0 | | | 0.071 | 0.765 | | 0 | | 0.062 | 0.673 | | 0 | | -0.074 | 0.092 | | 0 | |
| 1 | | | 0 | 0.765 | | 0.087 | | -17.942 | 0.673 | | 0.079 | | 21.473 | 0.092 | | 0.113 | |
| **z12**  0 | | | 0.068 | 0.838 | | 0.005 | | 0.276 | 0.799 | | 0.005 | | -0.199 | 0.039 | | 0.003 | |
| 1 | | | 0.004 | 0.838 | | 0.09 | | -5.195 | 0.799 | | 0.097 | | 3.748 | 0.039 | | 0.051 | |
| **z13**  0 | | | 0.071 | 0.776 | | 0.001 | | 0.138 | 0.712 | | 0.001 | | -0.135 | 0.065 | | 0.001 | |
| 1 | | | 0.001 | 0.776 | | 0.115 | | -11.348 | 0.712 | | 0.11 | | 11.114 | 0.065 | | 0.106 | |
| **z14** |  | 0 | 0.07 | | 0.959 | | 0.001 | 0.106 | | 0.958 | | 0.001 | -0.011 | | 0.001 | | 0 |
|  |  | 1 | 0.001 | | 0.959 | | 0.029 | -5.049 | | 0.958 | | 0.037 | 0.506 | | 0.001 | | 0 |

**II. Principal component analysis for risks and consequences of contracting COVID-19**

## Survey questions used

- (*prob_infectedatmarket*) Imagine that you go shopping in a crowded market for 2 hours. What is the probability that you will be infected with COVID-19 as a result? 1 Very unlikely/ 2 Unlikely/ 3 As likely as not/ 4 Likely/ 5 Very likely
- (*seriouslyill*) If you were infected with COVID-19, what is the likelihood that you would be so seriously ill that you would need to be hospitalised? 1 Very unlikely/ 2 Unlikely/ 3 As likely as not/ 4 Likely/ 5 Very likely
- (*die*) If you were infected with COVID-19, what is the likelihood that you would be so seriously ill that you would lose your life? 1 Very unlikely/ 2 Unlikely/ 3 As likely as not/ 4 Likely/5 Very likely

## Fear of COVID-19

| **Principal components/correlation** |  |  |  | **N=514** |
| --- | --- | --- | --- | --- |
| **Component** | **Eigenvalue** | **Difference** | **Proportion** | **Cumulative** |
| **Comp1** | 2.33675 | 1.80849 | 0.7789 | 0.7789 |
| **Comp2** | 0.528263 | 0.393277 | 0.1761 | 0.955 |
| **Comp3** | 0.134986 | . | 0.045 | 1 |

**Principal components**

**(Eigenvectors)**

| **Variable** | **Comp1** | **Comp2** | **Comp3** |
| --- | --- | --- | --- |
| **prob_infectedatmarket** | 0.5127 | 0.8533 | 0.0952 |
| **Seriously ill** | 0.6149 | -0.2875 | -0.7343 |
| **Die** | 0.5992 | -0.435 | 0.6721 |

# Appendix F: Reweighing sample using entropy balance

In the reweighted sample, condom use prevalence decreased from 77.2 percent (95% CI: 69.7-84.7 percent) in 2017 to 60.5 percent (95% CI: 52.6-68.5 percent) in 2020 and was statistically significant (p=0.0026).


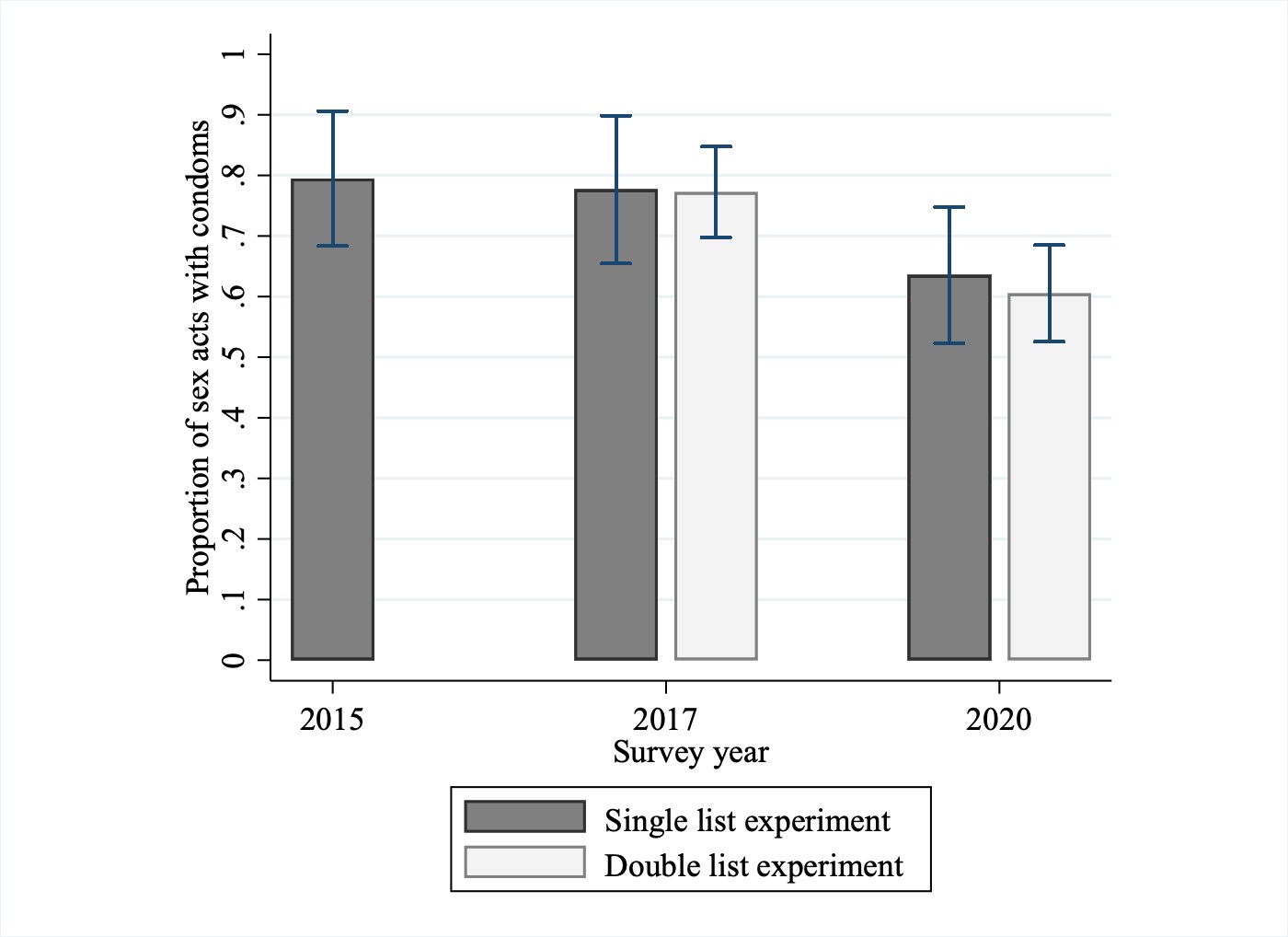


***Figure F.1: (Reweighted sample) Condom use prevalence estimates of FSWs in Dakar, Senegal***

## Table F.1: Sample moment balance before and after weighing (2015 (“Treat”) vs. 2017)

| **Table F.1** |  |  |  |  |  |  |
| --- | --- | --- | --- | --- | --- | --- |
|  |  |  |  |  |  |  |
| **Before: without weighting** | |  |  |  |  |  |
|  | ` |  |  |  |  |  |
|  |  | **Treat** |  |  | **Control** |  |
|  | **mean** | **variance** | **skewness** | **mean** | **variance** | **skewness** |
| **registered** | 0.4992 | 0.2504 | 0.0031 | 0.4980 | 0.2505 | 0.0078 |
| **age** | 35.84 | 85.58 | 0.2156 | 38.30 | 87.65 | 0.3913 |
| **2.marital** | 0.0107 | 0.0106 | 9.5020 | 0.0195 | 0.1919 | 6.9440 |
| **3.marital** | 0.6922 | 0.2134 | -0.8327 | 0.6895 | 0.2145 | -0.8189 |
| **4.marital** | 0.0521 | 0.0494 | 4.0320 | 0.0898 | 0.0819 | 2.8690 |
| **household** | 6.2480 | 27.65 | 1.2430 | 7.0530 | 29.39 | 1.5680 |
|  |  |  |  |  |  |  |
| **After: weight_2017 as the weighting variable** | | | |  |  |  |
|  | ` |  |  |  |  |  |
|  |  | **Treat** |  |  | **Control** |  |
|  | **mean** | **variance** | **skewness** | **mean** | **variance** | **skewness** |
| **registered** | 0.499 | 0.250 | 0.003 | 0.499 | 0.251 | 0.003 |
| **age** | 35.84 | 85.58 | 0.216 | 35.84 | 77.77 | 0.374 |
| **2.marital** | 0.107 | 0.011 | 9.502 | 0.011 | 0.011 | 9.488 |
| **3.marital** | 0.692 | 0.213 | -0.833 | 0.692 | 0.214 | -0.832 |
| **4.marital** | 0.052 | 0.049 | 4.032 | 0.052 | 0.050 | 4.027 |
| **household** | 6.248 | 27.65 | 1.243 | 6.250 | 23.63 | 1.662 |
|  |  |  |  |  |  |  |

## Table F.2: Sample moment balance before and after weighing (2015 (“Treat”) vs. 2020)

| **Table F.2** |  |  |  |  |  |  |
| --- | --- | --- | --- | --- | --- | --- |
|  |  |  |  |  |  |  |
| **Before: without weighting** | |  |  |  |  |  |
|  |  |  |  |  |  |  |
|  |  | **Treat** |  |  | **Control** |  |
|  | **mean** | **variance** | **skewness** | **mean** | **variance** | **skewness** |
| **registered** | 0.499 | 0.250 | 0.003 | 0.498 | 0.251 | 0.008 |
| **age** | 35.84 | 85.58 | 0.216 | 38.30 | 87.65 | 0.391 |
| **2.marital** | 0.011 | 0.011 | 9.502 | 0.020 | 0.192 | 6.944 |
| **3.marital** | 0.692 | 0.213 | -0.833 | 0.690 | 0.215 | -0.819 |
| **4.marital** | 0.052 | 0.049 | 4.032 | 0.090 | 0.082 | 2.869 |
| **household** | 6.248 | 27.65 | 1.243 | 7.053 | 29.39 | 1.568 |
|  |  |  |  |  |  |  |
| **After: weight_2020 as the weighting variable** | | | |  |  |  |
|  |  |  |  |  |  |  |
|  |  | **Treat** |  |  | **Control** |  |
|  | **mean** | **variance** | **skewness** | **mean** | **variance** | **skewness** |
| **registered** | 0.499 | 0.250 | 0.003 | 0.499 | 0.251 | 0.003 |
| **age** | 35.84 | 85.58 | 0.216 | 35.84 | 87.60 | 0.310 |
| **2.marital** | 0.011 | 0.011 | 9.502 | 0.011 | 0.011 | 9.504 |
| **3.marital** | 0.692 | 0.213 | -0.833 | 0.692 | 0.214 | -0.833 |
| **4.marital** | 0.052 | 0.049 | 4.032 | 0.052 | 0.050 | 4.030 |
| **household** | 6.248 | 27.65 | 1.243 | 6.249 | 23.63 | 1.318 |

## Table F.3: (Reweighted sample) Condom use prevalence by asset and debt status

**Double list experiment condom use prevalence Fall in condom use Fall in**

**estimate (%) (pp) condom**

### use w.r.t. 2017 (%)

**2017**

**2020**

|  | **N** | **mean** | **SE** | **N** | **mean** | **SE** |  |  |  |
| --- | --- | --- | --- | --- | --- | --- | --- | --- | --- |
|  |  |  |  |  | ***Panel A*** |  |  |  |  |
| **Asset status**  Asset poor | 213 | 85.1 | (6.1) | 280 | 57.3 | (5.5) | 27.8 | (p=0.0007) | 32.6 |
| Asset rich | 236 | 71.4 | (5.6) | 234 | 64.2 | (6.1) | 7.3 | (p=0.36) | 10.2 |
| *Difference* |  |  |  |  |  |  | 20.5 | (p=0.078) |  |
|  |  |  |  |  | ***Panel B*** |  |  |  |  |
| **Debt status**  Indebted | 263 | 79.5 | (5.7) | 280 | 69.6 | (5.3) | 9.9 | (p=0.19) | 12.4 |
| Not indebted | 250 | 75.0 | (5.1) | 234 | 51.1 | (6.2) | 23.9 | (p=0.0027) | 31.9 |
| *Difference* |  |  |  |  |  |  | -14.0 | (p=0.20) |  |
|  |  |  |  |  | ***Panel C*** |  |  |  |  |
| **Asset status X Deb**  ***Asset poor***  Indebted | **t statu**    110 | **s**    90.6 | 9.2 | 181 | 64.9 | 6.9 | 25.6 | (p=0.022) | 28.3 |
| Not indebted | 103 | 79.8 | 8.0 | 99 | 44.9 | 8.8 | 34.9 | (p=0.0031) | 43.7 |
| *Difference* |  |  |  |  |  |  | -9.3 | (p=0.56) |  |
| ***Asset rich***  Indebted | 115 | 72.0 | 8.4 | 99 | 78.3 | 8.0 | -6.3 | (p=0.58) | -8.7 |
| Not indebted | 121 | 70.9 | 7.5 | 135 | 55.4 | 8.5 | 15.5 | (p=0.17) | 21.8 |
| *Difference* |  |  |  |  |  |  | -21.8 | (p=0.17) |  |

Notes: Standard errors (SE) were clustered by respondent. The double list experiment was implemented only in 2017 and 2020. Debt status was elicited only in 2017 and 2020. Asset status was elicited only in 2015 and 2020. Asset status in 2017 was first filled with asset status in 2020, and then by asset status in 2015.

# Appendix G: Other mechanisms

We elicited questions related to respondents’ perception of COVID-19 and its effect on their behaviour only in 2020. Consequently, the analyses of other mechanisms have to be restricted to the sample of repeat active FSWs. This presents two issues. First, the list experiment is less likely to have statistical significance with a smaller sample size. Second, the characteristics of this restricted sample are fundamentally different from overall sample. For example, repeat participants will more likely be older women than newer recruits. Therefore there could be limitations of extending the conclusions of the analysis on the restricted sample to the whole sample. Furthermore, the condom use prevalence change within this sample is 6.0 percentage points (7.8 percent), which is much smaller than that of the overall sample. Therefore, an assumption for the results in this section to have external validity is that this restricted sample should experience similar behavioural responses to COVID-19 as the overall sample. We first test this by repeating the analyses on asset and debt status. We then move on to cover mechanisms related to a) potential condom supply restrictions, b) substitutions between risks of contracting COVID-19 and HIV/STIs, and c) shifted to seeing more regular clients because of COVID-19.

**Note**: There are also other important mechanisms that we do not have any data on, such as reduced risk from lower client numbers permit more risk-taking in terms of condom use, and clients having greater bargaining power to pressure FSWs to forgo protection.

## G.1 Asset and debt status

We see a similar trend in the restricted sample as the whole sample. The point estimates of condom use prevalence decline was higher amongst the asset poor (11.3 vs. 1.4 percent) and the debt-free (14.9 vs. 0.3 percent) (Table F.1: Panel A and B). However, despite the high magnitudes, the declines in condom use prevalence across the two years for the asset poor and the debt-free were no longer statistically significant, unlike in the whole sample.

## G.2 Potential condom supply restrictions

Registered female sex workers are required to attend monthly clinic visits, which avail them to the opportunity to obtain free condoms from the clinics. In 2020, they reported on a 5-point Likert scale how much they reduced their monthly clinic visits because of COVID-19. Amongst those who reported reducing these visits, condom use was high (over 90 percent) and was almost the same for both 2017 and 2020 (Table F.1: Panel C). The high condom rates amongst this group in 2020 suggest that condom supply in the broader environment is unlikely to have faced a major longer-term disruption.

## G.3 Substitution between risks of contracting COVID-19 and HIV/STIs

Seeing more clients could also expose FSWs to the risk of contracting COVID-19. FSWs who are keen in avoiding health risks from COVID-19 may prefer to have fewer clients, and instead, take on higher risks of contracting HIV/STIs in order to maintain their income. However, at the same time, fear of diseases could be positively correlated. This would reduce the probability of this type of response.

We look at how condom use decline differed between FSWs with a higher fear of COVID-19, versus those with a lower fear of COVID-19. Respondents were asked about (a) their perceived risks of contracting COVID-19 if they were a crowded market for two hours, as well as (b) if they were to contract COVID-19, how likely they would be severely ill, or (c) die. These questions used a 5-point Likert-type scale, with 1 representing very unlikely and 5 representing very likely. We reduce the dimensionality of these three questions via principal component analysis (Appendix E). We used the first principle component to represent latent fear of COVID-19. We grouped the respondents as having a high fear of COVID-19 if the predicted values of their first principal component were more than that of the median.

The first principal component explained 77.9 percent of the variance in the three questions on the likelihood and consequences of contracting COVID-19 (Appendix E.II). While none of the condom use declines were statistically significant in both subgroups with differing levels of fear of COVID-19, the point estimate of the condom use decline was larger amongst those who have low fear of COVID-19 (10.1 percentage points, p=0.26) than amongst those with a high fear of COVID-19 (2.1 percentage points, p=0.84) (Table F.1: Panel D). Therefore, we find no evidence that a fear of COVID-19 increases the propensity for FSWs to take more sexual risks.

## G.4 Shift to seeing more regular clients (risk compensation)

COVID-19 and its mitigating measures has likely made it more difficult for sex workers to find new clients. Therefore, we expect FSWs to shift towards a more regular clientele. Indeed, the data shows that 39.7 percent of FSWs reported seeing more regular customers because of COVID-19. In contrast, 5.7 percent of FSWs reported seeing more casual customers.

Regular clients are typically considered as less risky (Ito et al., 2018), and therefore, a shift towards regular clients may have reduced the perceived necessity to use condoms. Robinson and Yeh (2011) also mentioned women in Kenya were more likely to have unprotected sex with regular clients than casual clients, presumably due to greater awareness of the HIV/STI risks of clients they know better. FSWs who shift towards regular clients may also more likely be those who were originally soliciting outdoors, and hence, might have seen a larger drop in income. Both of these increase the likelihood that FSWs who reported a shift towards regular clients because of COVID-19 saw a steeper decline in condom use prevalence than those who did not.

We did not see this in the data. Condom use declines were statistically insignificant both amongst FSWs who reported they have shifted to regular clients due to COVID-19, and amongst those who did not (Table F.1: Panel E). The point estimates of the condom use declines were similar in both subgroups (6.0 percentage points, p=0.58 versus 8.8 percentage points, p=0.31), and was less steep for the group for which we expected to see a steeper decline.

**Table G.1: Condom use prevalence of subgroups in restricted sample**  **N Double list experiment condom Fall in condom Fall in**

**(2017/ use prevalence estimate (%) use condom use**

### 2020) 2017 2020 (pp) w.r.t. 2017 mean SE mean SE ^(%)^

|  |  | | |  | ***Panel A*** |  |  |  |  |
| --- | --- | --- | --- | --- | --- | --- | --- | --- | --- |
| **Asset status**  Asset poor | 147 81.6 | | | 6.9 | 70.3 | 6.8 | 11.3 | (p=0.25) | 13.8 |
| Asset rich | 139 73.0 | | | 6.3 | 71.6 | 7.6 | 1.4 | (p=0.88) | 2.0 |
| *Difference* |  | | |  |  |  | 9.8 | (p=0.47) |  |
|  |  | | |  | ***Panel B*** |  |  |  |  |
| **Debt status**  Indebted | 141/132 80.5 | | | 7.2 | 80.2 | 6.9 | 0.3 | (p=0.98) | 0.4 |
| Not indebted | 145/154 73.2 | | | 6.2 | 58.3 | 7.3 | 14.9 | (p=0.10) | 20.4 |
| *Difference* |  | | |  |  |  | -14.6 | (p=0.27) |  |
|  |  | | |  | ***Panel C*** |  |  |  |  |
| **Registered FSWs reduced m**  Not reduced 60 | | **onthly cli**  90.1 | **nic visits**  (9.7) 72.2 | | | (9.4) | 17.9 | (p=  0.19) | 19.9 |
| Reduced 78 | | 90.8 | (8.7) 90.6 | | | (11.3) | 0.3 | (p=0.98) | 0.3 |
| *Difference* | |  |  | | |  | 17.7 | (p=0.36) |  |
|  | |  | ***Panel D*** | | |  |  |  |  |
| **Fear of COVID-19**  High fear 119 | | 78.1 | (7.8) 76.0 | | | (7.4) | 2.1 | (p=0.84) | 2.6 |
| Low fear 167 | | 76.3 | (5.9) 66.2 | | | (6.1) | 10.1 | (p=0.26) | 13.2 |
| *Difference* | |  |  | | |  | -8.0 | (p=0.55) |  |
|  | |  | ***Panel E*** | | |  |  |  |  |
| **Reported shift to regular clients due to COVID-19**  Shifted 112 78.6 (8.0) 72.6 | | | | | | (7.9) | 6.0 | (p=0.58) | 7.6 |
| Not shifted 174 75.9 (5.9) 67.1 | | | | | | (6.4) | 8.8 | (p=0.31) | 11.6 |
| *Difference* | | | | | |  | -2.8 | (p=0.84) |  |

# Appendix H: Attrition

***Table H.1*: *Determinants of attrition in 2017***

**Variables**

**Participant resurveyed & still in**

**sex work in 2017**

**Attrited participant**

**Mean Diff**

|  | **N** | **mean** | **N** | **mean** |  |
| --- | --- | --- | --- | --- | --- |
| Age | 378 | 36.72 | 275 | 34.63 | 2.084*** |
| Registered FSW (Prop.) | 378 | 0.508 | 275 | 0.487 | 0.0210 |
| Never married (Prop.) | 378 | 0.225 | 275 | 0.273 | -0.0480 |
| Married (Prop.) | 378 | 0.0110 | 275 | 0.0110 | 0 |
| Divorced (Prop.) | 378 | 0.706 | 275 | 0.673 | 0.0340 |
| Widowed (Prop.) | 378 | 0.0580 | 275 | 0.0440 | 0.0150 |
| Sex work earnings in a month ('000 CFAF) | 378 | 135.5 | 273 | 133.6 | 1.978 |
| Clients in a week (No.) | 378 | 6.725 | 274 | 6.234 | 0.491 |
| Average price of last two clients ('000 CFAF) | 355 | 17.07 | 267 | 16.29 | 0.778 |
| Total household expenses in last 30 days ('000 CFAF) | 378 | 341.0 | 275 | 314.7 | 26.33 |
| Savings in last 30 days ('000 CFAF) | 374 | 16.73 | 273 | 15.13 | 1.604 |
| Share of occasional clients (Prop.) | 370 | 0.379 | 267 | 0.418 | -0.0390 |
| At least one outdoor solicitation method (Prop.) | 376 | 0.713 | 274 | 0.657 | 0.0560 |
| At least one outdoor place of sex (Prop.) | 376 | 0.380 | 274 | 0.369 | 0.0120 |

**Note:** *p<0.1, **p<0.05, ***p<0.01.

## *Table H.2*: *Determinants of attrition in 2020*

**Variables**

**Participant resurveyed & still in**

**sex work in 2020**

**Attrited participant**

**Mean Diff**

|  | **N** | **mean** | **N** | **mean** |  |
| --- | --- | --- | --- | --- | --- |
| Age | 285 | 38.80 | 227 | 37.67 | 1.134 |
| Registered FSW (Prop.) | 285 | 0.512 | 227 | 0.480 | 0.0320 |
| Never married (Prop.) | 285 | 0.179 | 227 | 0.229 | -0.0500 |
| Married (Prop.) | 285 | 0.0140 | 227 | 0.0260 | -0.0120 |
| Divorced (Prop.) | 285 | 0.723 | 227 | 0.648 | 0.075* |
| Widowed (Prop.) | 285 | 0.0840 | 227 | 0.0970 | -0.0130 |
| Sex work earnings in a month ('000 CFAF) | 282 | 122.6 | 225 | 133.8 | -11.21 |
| Clients in a week (No.) | 285 | 8.877 | 227 | 7.727 | 1.150 |
| Average price of last two clients ('000 CFAF) | 285 | 14.52 | 227 | 19.11 | -4.597 |
| Total household expenses in last 30 days ('000 CFAF) | 285 | 319.5 | 227 | 348.8 | -29.39 |
| Savings in last 30 days ('000 CFAF) | 285 | 21.53 | 227 | 21.95 | -0.415 |
| Share of occasional clients (Prop.) | 279 | 0.331 | 225 | 0.306 | 0.0250 |
| At least one outdoor solicitation method (Prop.) | 285 | 0.561 | 227 | 0.502 | 0.0590 |
| At least one outdoor place of sex (Prop.) | 285 | 0.642 | 227 | 0.542 | 0.100** |

**Note:** In this appendix, we define attrition with respect to the immediate previous wave’s survey participation and “new participant” as not having participated in the immediate previous survey wave. Hence, a participant who participated in the 2015 and 2020 surveys would by construct be considered as an attrited participant in 2017, and a new participant in 2020. *p<0.1, **p<0.05, ***p<0.01.

# Appendix I: Adjusting for Tabaski – a concomitant economic shock

It is important to account for other factors that may also have an impact on condom use prevalence, but is not related to COVID-19. We do a robustness check to see whether our results still hold after taking into account one such confounder.

In 2017 and 2020, the surveys were administered close to the start of Tabaski- an important annual festival in Senegal. It is customary for individuals to buy expensive goats or sheep for this celebration^[[1]](#footnote-1)^, which could impose an additional consumption shock on our respondents. Therefore, the fall in condom use prevalence between 2017 and 2020 could include both the effect of COVID-19 on condom use prevalence during non-festival times, as well as the exacerbating effects the COVID-19 pandemic has on an existing annual consumption shock. Both of these effects would be of interest especially when modelling HIV/STI transmission risks, but with our data, we would be unable to disentangle these two effects.

The last day of data collection was eight and four days before Tabaski in 2017 and 2020 respectively. Hence, sex acts between four to seven days before Tabaski were absent in the 2017 data, but were present in the 2020 data. Aligning the period of exposure to Tabaski could be important as over half of those who reported buying an animal for the 2017 Tabaski did so in the last two days before Tabaski. Hence, part of the fall in condom use prevalence between 2017 and 2020 could potentially be attributable to the inclusion of more sex acts that were exposed to the effects of Tabaski.

Our robustness check specification involves omitting sex acts that were performed four to seven days before Tabaski (n=37; 7.2% of 2020 sample). We also omitted sex acts occurring more than 365 days ago (n=31; 6.0% of 2017 sample; n=9; 1.8% of 2020 sample) as these were likely to be errors given the sample consisted of active FSWs.

A point to note is that the adjusted 2020 sample is more likely to have sex acts further away from Tabaski than the 2017 sample (Figure I.1). Therefore, the change due to this modification could be overstated. In addition, the sample replenishment occurred towards the end of the survey week. Hence, 97.3% of the respondents whose sex acts were omitted in this robustness check were first-time respondents and were on average 6.5 years younger than the rest of the sample in 2020. This could in itself introduce new biases. Nonetheless, it is still a useful check to do to see whether the conclusions we made in our main analysis are robust.


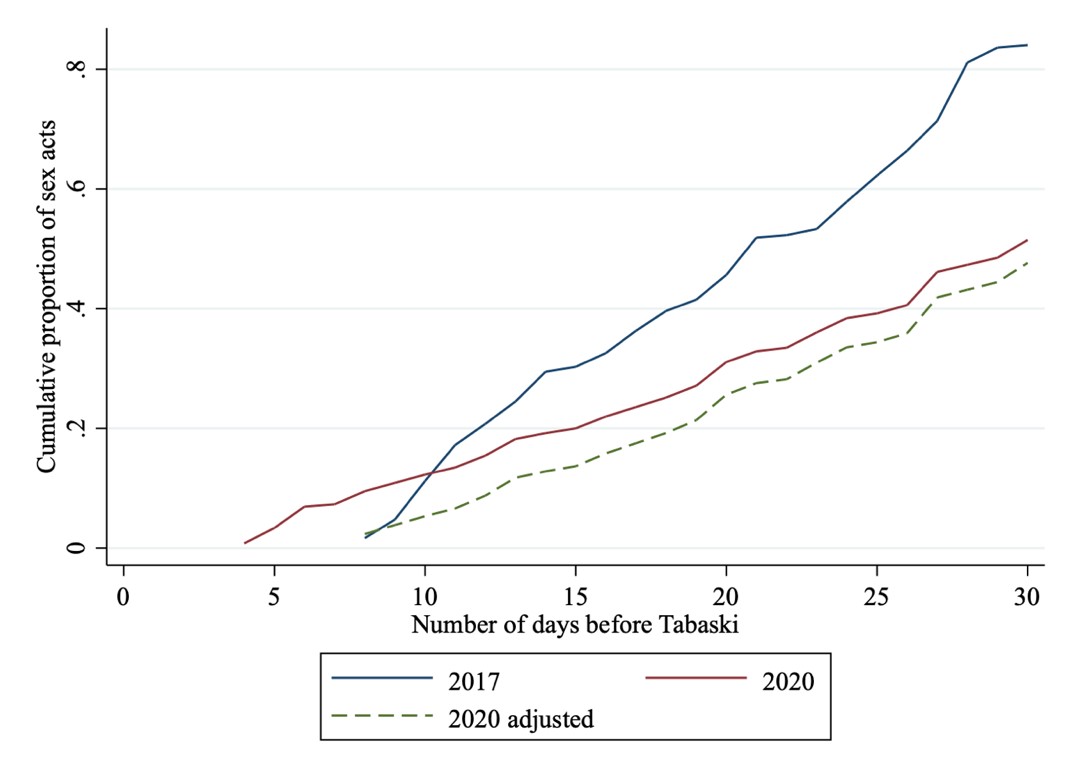


## *Figure I.1*: Cumulative distribution of sex acts N days before Tabaski

The adjusted double list experiment condom use prevalence estimates were 78.8 percent (95% CI: 71.2-86.5 percent) and 68.4 percent (95% CI: 60.6-76.3 percent) in 2017 and 2020 respectively. This decline of 10.4 percentage points (p=0.061), corresponds to a 13.2 percent fall in condom use prevalence and a 49.1 percent increase in unprotected sex- approximately four-fifths of the impact seen in the main analysis. Results on the subgroup heterogeneities in the decline of condom use prevalence remain consistent with the key interpretations and discussions in the main analysis (Table I.1).

***Table I.1*: Condom use prevalence by asset and debt status in restricted sample**

**Double list experiment condom use prevalence Fall in condom Fall in**

**estimate (%) use condom use**

### (pp) w.r.t. 2017 N SE N SE ^(%)^

**2017**

**2020**

**mean**

**mean**

|  |  | |  |  | ***Panel A*** |  |  |  |  |
| --- | --- | --- | --- | --- | --- | --- | --- | --- | --- |
| **Asset status**  Asset poor | 236 84.2 | | (5.7) | 224 | 65.3 | (5.7) | 19.7 | (p=0.014) | 23.4 |
| Asset rich | 213 73.8 | | (5.6) | 253 | 74.1 | (5.6) | -0.3 | (p=0.97) | -0.4 |
| *Difference* |  | |  |  |  |  | 20.0 | (p=0.070) |  |
|  |  | |  |  | ***Panel B*** |  |  |  |  |
| **Debt status**  Indebted | 263 80.9 | | (5.3) | 258 | 76.4 | (5.5) | 4.5 | (p=0.55) | 5.6 |
| Not indebted | 250 75.4 | | (5.2) | 219 | 59.8 | (5.7) | 15.6 | (p=0.039) | 20.7 |
| *Difference* |  | |  |  |  |  | -11.1 | (p=0.29) |  |
|  |  | |  |  | ***Panel C*** |  |  |  |  |
| **Asset status X De**  ***Asset poor***  Indebted | **bt status**    110 | 90.4 | 8.2 | 161 | 74.7 | 6.8 | 15.7 | (p=0.13) | 17.4 |
| Not indebted | 103 | 77.7 | 8.2 | 92 | 46.7 | 8.9 | 31.1 | (p=0.009) | 39.9 |
| *Difference* |  |  |  |  |  |  | -15.3 | (p=0.32) |  |
| ***Asset rich***  Indebted | 115 | 74.8 | 8.0 | 97 | 79.8 | 8.4 | -4.9 | (p=0.67) | -6.6 |
| Not indebted | 121 | 72.8 | 7.8 | 127 | 69.4 | 7.3 | 3.3 | (p=0.75) | 4.6 |
| *Difference* |  |  |  |  |  |  | -8.2 | (p=0.60) |  |

# References

1. Ito, S., Lépine, A., & Treibich, C. (2018). The effect of sex work regulation on health and well-being of sex workers: Evidence from Senegal. *Health economics*, *27*(11), 16271652.
2. Robinson, J., & Yeh, E. (2011). Transactional sex as a response to risk in Western

Kenya. *American Economic Journal: Applied Economics*, *3*(1), 35-64.

1. 82.9% of our sample in 2020 intend to celebrate Tabaski in 2020. Amongst these respondents, 58.6% of the respondents intended to contribute towards purchasing an animal in 2020. [↑](#footnote-ref-1)
